# Investigation of Constitutive Models for Pressure Monitoring of Viscoelastic–Hyperelastic Composite Structures

**DOI:** 10.3390/polym17050647

**Published:** 2025-02-28

**Authors:** Lijia Ai, Peng Li, Hongwei Yuan, Chunrong Tian, Xiaolian Qiang, Tao Fu

**Affiliations:** 1 Institute of Chemical Materials, China Academy of Engineering Physics, Mianyang 621900, China; 20181985@s.hlju.edu.cn (L.A.); yuanhw@caep.cn (H.Y.); tianchr_icm@caep.cn (C.T.); qiang.xiaolian@caep.cn (X.Q.); 2 College of Physical Science and Technology, Heilongjiang University, Harbin 150080, China; lipenghit@hlju.edu.cn

**Keywords:** rigid polyurethane foam (RPUF), silicone rubber foam (SRF), constitutive model, quasi-static compression, pressure monitoring

## Abstract

To ensure ammunition safety, a protective structure and pressure detection system are essential; however, there is a lack of an accurate constitutive model to describe the mechanical response characteristics of protective structures composed of various polymer materials. In this work, a constitutive model for the composite structure based on the superposition principle is successfully constructed derived from the quasi-static compression behavior of rigid polyurethane foam (RPUF), silicone rubber foam (SRF), and flexible pressure sensors (FPSs) through experimental investigations. The constitutive model accurately reflects the influence of each type of polymer foam on the mechanical performance of composite structures, underscoring the significance of thickness ratios. Test results within the temperature range of 25 °C to 55 °C validate the model’s accuracy, with an average fitting error of 8.6%. Furthermore, a multi-channel pressure detection system has been integrated into the composite structure. Under conditions of out-of-plane loads ranging from 0 to 10 kilonewtons, the accuracy of the pressure monitoring system, adjusted using the constructed model, has improved by 16%. The constitutive model and the pressure sensing system effectively predict the mechanical properties of the protective structure and enable real-time force state monitoring, which is crucial for ammunition safety and has broader applications for safeguarding other objects.

## 1. Introduction

Ammunition is typically composed of energetic particles, which may experience minor impact forces due to collisions, vibrations, or drops during activities like storage, handling, or transportation [1,2]. These low-level impacts can cause subtle damage, which may jeopardize the operational reliability of the ammunition and could even lead to unintended detonation [3,4,5]. Consequently, the development of effective ammunition protection structures and the establishment of sensitive condition monitoring systems hold significant research importance [6,7].

Rigid polyurethane foam (RPUF) is a viscoelastic polymer material made by mixing specific proportions of isocyanate, polyol, and various additives [8]. Due to its excellent cushioning properties, cost-effectiveness, and ease of molding, it is extensively utilized in protective packaging for critical items, and it has emerged as the preferred material for ammunition protection systems [9,10]. However, the rigid nature of RPUF creates slight gaps at the interface with the ammunition [11]. Silicone rubber foam (SRF), a common hyperelastic material, is widely used in engineered systems as impact-absorbing support cushions owing to its superior thermal stability, excellent energy absorption capabilities, and good damping characteristics. Consequently, a layer of SRF is often incorporated within the RPUF protective structural components to safeguard the ammunition, and, compared to single-layer cushioning systems, the RPUF–SRF composite structure enhances the range of resistance to external impact stresses [12,13]. Meanwhile, it is essential to implement a monitoring system that facilitates real-time evaluations of the stress conditions on the surface of the ammunition, with the objective of generating safety alerts [14]. Compared to other pressure sensing systems, flexible pressure sensing systems (FPSs) exhibit superior performance for long-term monitoring applications, making them particularly suitable for specialized environments, such as ammunition safety performance monitoring [15]. Significantly, the monitoring results from the pressure sensing system do not align with the external impact load only [16]. It is suggested that the presence of energy-absorbing buffer structures would generate a gradual attenuation of external loads when they are transmitted to the surface of the ammunition [17]. Therefore, to assess the protective efficacy of the RPUF–SRF composite structure and ensure the precision of the pressure sensing system’s monitoring, it is crucial to investigate the constitutive model of the RPUF–SRF–FPS composite structure [18].

Constitutive models of multilayered cushioning material composite structures under quasi-static loading conditions have been developed in recent years [19,20]. For example, Merzhievskii and Voronin [21] constructed a Maxwellian elastoplastic body to describe the behavior of polymethyl metacrylate under loading. Such an approach allows for obtaining a unified mathematical description of all physical states of polymers. Popova et al. [22] used Maxwell relaxation models for describing the viscoelasticity of both materials in polymethylmethacrylate (PMMA) and aluminum. The approach, given its complexity and accuracy, lies between the simple relaxation models and the complete-dislocation-based ones. Qian et al. [23] developed a novel layered foam composite structure varying from two to five layers. Their results explored the effect of the layer count on the mechanical properties of the foam composite structure, with experimental outcomes indicating that the two-layer foam composite structure demonstrates the greatest ductility relative to other configurations. Çıbıkçı et al. [24] introduced an innovative foam composite structure composed of closed-cell aluminum foam and expanded polystyrene (EPS) foam. Experimental evaluations of its compressive properties demonstrated a significant enhancement in both compressive strength and the damping ratio compared to single-layer aluminum foam. Salehi et al. [25] examined the compressive behavior and energy absorption potential of discrete layered foam-filled tubes constructed from closed-cell zinc foam, aluminum foam, and A356 alloy foam. The results reveal that the multilayer foam-filled tubes display multiple quasi-static responses and a progressive increase in stress through a sequential collapse, beginning with the low-density components. Furthermore, the dynamic response of the gradient structures was forecasted using the established model. Gupta et al. [26] utilized a three-parameter empirical constitutive model to analyze the influence of various density combinations of RPUF on their mechanical properties and energy absorption capabilities. Their findings suggest that the stress–strain curve response can be tailored by adjusting the density of the RPUF. Yang et al. [27] fabricated foam-filled multi-layered hybrid composite graded lattice sandwich panels (MHCGLSPs) with different configurations and investigated their dynamic response characteristics experimentally and numerically. The influences of filled foam and the graded arrangement on the crushing behavior of the panels are depicted. Furthermore, the numerical results obtained based on the Hashin failure criterion [28] and the Johnson–Cook model [29] are in good agreement with the experimental results.

Although a series of studies have been conducted on the properties of monolayer foam configurations, which can greatly affect the mechanical performance of the entire structure [30,31], previous studies have predominantly focused on layered structures composed of polymers with similar material properties, such as viscoelasticity. There remains a need for further investigation into the mechanical properties of layered structures utilizing polymers with differing material characteristics. Additionally, it is essential to explore the impact of layer dimensions on the compressive strength of composite material structures. Therefore, developing a model that accurately characterizes the mechanical properties of viscoelastic–hyperelastic composite structures is of considerable research significance.

Herein, the compressive mechanical behavior of various single-layer structures is investigated through quasi-static compression tests performed within a temperature range of 25–55 °C. The outcome is a function that characterizes the variation of RPUF material properties as a function of thickness. A constitutive model is developed based on the principle of superposition utilizing piecewise functions. The stress–strain behavior of RPUF–SRF–FPS composite structures with differing dimensions is analyzed, alongside an examination of the internal ballast force propagation characteristics within these composite structures. Additionally, a real-time pressure monitoring system for ammunition surfaces is established to evaluate the stability of the pressure monitoring system across a temperature range of −20 °C to 70 °C. Final validation is achieved through the application results of the actual pressure monitoring system. The findings of this research are intended to enhance protective structure performance and improve ammunition safety monitoring systems.

## 2. Materials and Methods

### 2.1. Materials

RPUF [32] with a density of 500 kg/m^3^ was synthesized using polyols and methylene diphenyl isocyanate (MDI). The RPUF specimens are cylindrical, cut from a single piece of RPUF, and processed using a lathe during the cutting process to prevent the formation of oxide scale on the surface. They are manufactured according to the guidelines of ASTM D1621 (Table 1).

The SRF [33] was formed from silica-reinforced silicone rubber gum containing hexane. The foams were processed into cylindrical samples with diameters of 60 mm and heights of 1.8 mm for testing.

The FPS [34] was produced by Guangzhou Puhui Responsibility Co., Ltd. (Guangzhou, China). This sensor has a thickness of only 0.3 mm and a measurement range of up to 8 MPa. Based on the novel hierarchical microstructures, the FPS exhibits a sensitivity of 15.4 kPa^−1^. The sensing element is a square with dimensions of 14 × 14 mm^2^, and the sensing material consists of reduced graphene oxide (rGO) and Ag nanowires (AgNWs) integrated with a real-time data acquisition system.

### 2.2. Experimental Methods

**Figure 1:**
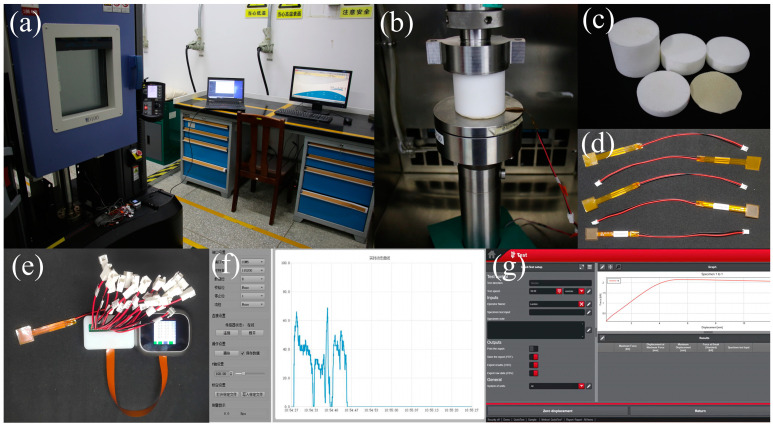
Experimental equipment. (**a**) Experimental setup. (**b**) Quasi-static compression experiments. (**c**) Experimental materials RPUF and SRF. (**d**) FPS. (**e**) FPS monitoring system. (**f**) Acquisition module. (**g**) Universal testing machine measurement system.

Figure 1 illustrates that the quasi-static uniaxial compression tests of the RPUF–SRF–FPS composite structure were conducted using the universal testing machine. The experimental procedure employed a stepwise loading approach, systematically increasing the load from 0 to 4 kN. By setting the loading speed, the stress–strain curves at strain rates of 0.5 mm/min were obtained. During the loading process, the compressive load was acquired using a high-precision force sensor mounted on the beam, while the testing machine measured the loading displacement by recording the distance the beam moved.

### 2.3. Stability of the FPS

**Figure 2:**
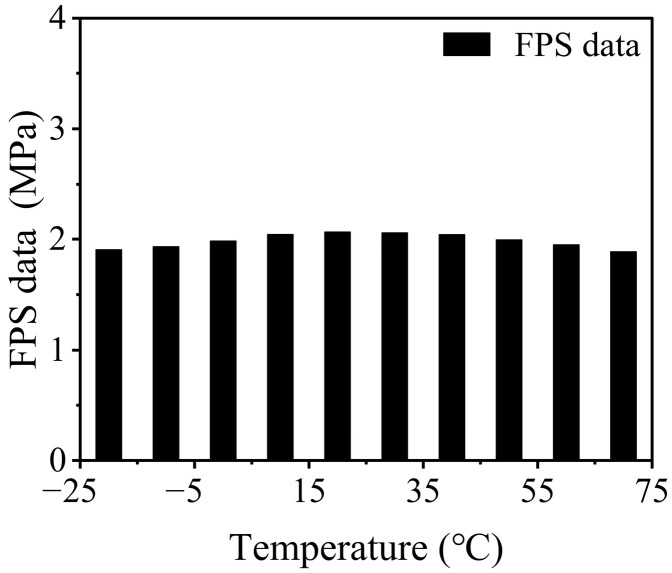
The 2 MPa load pressure sensor indicates a drift curve with temperature.

In order to thoroughly investigate the stability characteristics of the pressure sensor, we conducted a series of experiments to evaluate its response values under a range of temperature conditions over a specified duration. Regarding the influence of temperature, the response value of the pressure sensor to the 2 MPa loading pressure was measured at various temperatures. We positioned the FPS specimen between the grips of the universal testing machine, applying a constant load of 0.4 kN. We utilized a temperature control chamber to elevate the environmental temperature of the FPS from −20 °C to 70 °C while observing and recording the output data of the FPS. The monitored output data recorded by the system represent the pressure, defined as *P* = *F*/*A*_1_, where *P* is the FPS output data, *F* is the constant loading force of 0.4 kN, and *A*_1_ is the area of the FPS sensing unit, which measures 14 × 14 mm^2^. As shown in Figure 2, the response values of the pressure sensor exhibited only minor fluctuations when subjected to temperature conditions ranging from −20 °C to 70 °C. This observation indicates a remarkable level of stability of the pressure sensor when exposed to diverse temperature scenarios, thereby affirming its reliability for applications in varying thermal settings.

## 3. Constitutive Model

### 3.1. Model Description

**Figure 3:**
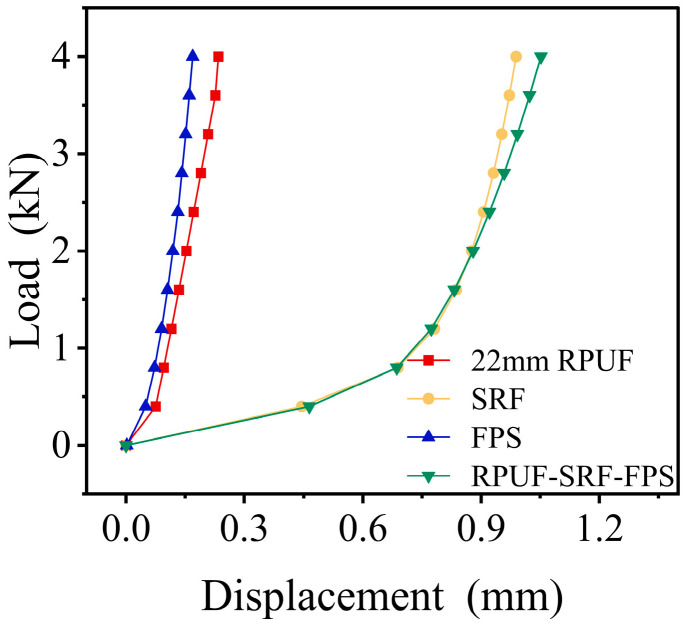
Displacement–load curves of each single-layer structure and the 22 mm RPUF composite structure.

Experiments were carried out using a universal testing machine by controlling the experimental load through the high-precision pressure sensors integrated into the material testing apparatus. The displacement–load curves for each individual layer structure and the composite structures can be obtained through quasi-static uniaxial compression tests, as illustrated in Figure 3. The experimental results clearly indicate that the mechanical behavior of the composite structure manifests as a superposition of individual monolayer structures, significantly influenced by the properties of the hyperelastic material SRF. Furthermore, within the specified load range, the RPUF remains in the initial linear elastic region.

**Figure 4:**
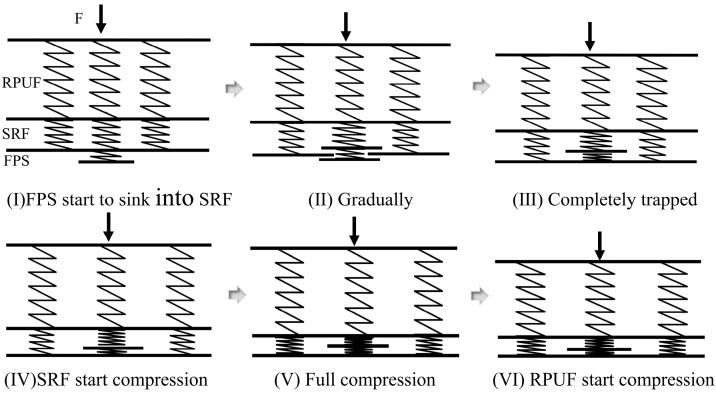
The deformation process of a composite structure spring model under an out-of-plane compressive load.

Consequently, the compression process of the RPUF–SRF–FPS composite structure under external loading can be modeled using multiple series of connected spring systems [35], as illustrated in Figure 4.

In the preliminary stages (I) to (III), it is assumed that the materials between the layers of the composite structure are fully bonded, satisfying the strain compatibility conditions. The overall deformation of the composite structure can be understood as the sum of the deformations of RPUF, SRF, and FPS. During this phase, the forces exerted on each distinct layer are equivalent, and the total deformation of the composite structure is characterized by the cumulative deformations of each individual layer:

(1) $$\Delta x=\frac{F}{k_{1}}+\frac{F}{k_{2}}+\frac{F}{k_{3}}$$

where *F* is the external loading force, Δx represents the total deformation of the composite structure, *k*_1_, *k*_2_, and *k*_3_ are the stiffness of RPUF, SRF and FPS, and

(2) $$k=\frac{EA_{1}}{d}$$

where *A*_1_ is the cross-sectional area of the FPS, *E* is the elastic modulus, and *d* representing the material thickness.

By substituting Equation (2) into Equation (1), one can derive

(3) $$\Delta x=\frac{Fd_{1}}{E_{1}A_{1}}+\frac{Fd_{2}}{E_{2}A_{1}}+\frac{Fd_{3}}{E_{3}A_{1}}$$

where *E*_1_, *E*_2_, and *E*_3_ represent the elastic moduli of RPUF, SRF, and FPS. And, *d*_1_, *d*_2_, and *d*_3_ are the thicknesses of the RPUF, SRF, and FPS.

In stages (IV) to (VI), the FPS is entirely immersed in the SRF, and the strain components are equal across all sections. The composite structure forces *F* are decomposed into RPUF–SRF–FPS partial force *F*_1_ and RPIF–SRF partial force *F*_2_:

(4) $$\Delta x=\frac{F_{1}}{k_{1}}+\frac{F_{1}}{k_{2}}+\frac{F_{1}}{k_{3}}=\frac{F_{2}}{k_{1}^{\prime }}+\frac{F_{2}}{k_{2}^{\prime }}$$

where $k_{1}^{\prime }=E_{1}A_{2}/d_{1}$, $k_{2}^{\prime }=E_{2}A_{2}/d_{2}$, $F=F_{1}+F_{2}$. The total load-bearing area of the composite structure is $A=A_{1}+A_{2}$, while *A*_2_ represents the load-bearing area of the RPUF–SRF section. By substituting into Equation (4), one can derive

(5) $$\Delta x=\frac{F_{1}d_{1}}{E_{1}A_{1}}+\frac{F_{1}d_{2}}{E_{2}A_{1}}+\frac{F_{1}d_{3}}{E_{3}A_{1}}=\frac{F_{2}d_{1}}{E_{1}A_{2}}+\frac{F_{2}d_{2}}{E_{2}A_{2}}$$

When the strain ε=Δx/D, the stress σ=F/A, and the composite structure thickness $D=d_{1}+d_{2}+d_{3}$, then the constitutive model of the composite structure at each stage can be expressed as Equation (6) [36]:

(6) $$\left\{\begin{array}{cc} \sigma_{1}=\frac{\varepsilon }{\alpha +\beta +\gamma } & \;\;\;\;0\leq \varepsilon <\frac{0.3}{D}\\ \sigma_{2}=\left(\varepsilon -\frac{0.3}{D}\right)\left[\frac{\alpha +\beta +\frac{A_{2}}{A}\gamma }{\left(\alpha +\beta +\gamma \right)\left(\alpha +\beta \right)}\right]+\sigma_{1} & \frac{0.3}{D}\leq \varepsilon \leq 0.1 \end{array}\right.$$

where $\alpha =d_{1}/DE_{1}(d_{1},T)$, $\beta =d_{2}/DE_{2}(T)$, and $\gamma =d_{3}/DE_{3}(T)$.

Equation (6) describes the quasi-static compressive stress of the RPUF–SRF–FPS composite structure. In order to forecast the quasi-static stress–strain behavior of multi-layered structures, the model integrates the stress–strain characteristics of each individual material layer and applies the superposition principle applicable to linear elastic materials. The dimensions of each layer are considered by normalizing the stress and strain values in relation to the layer thickness and the cross-sectional area.

### 3.2. Parameter Determination

**Figure 5:**
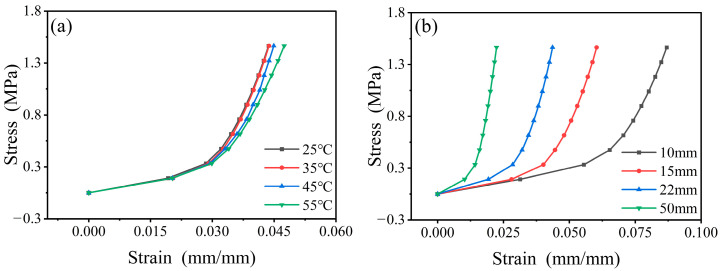
Composite structure stress–strain curves. (**a**) The 22 mm RPUF composite structure stress–strain curves at different temperatures. (**b**) Stress–strain curves of composite structures with different RPUF thicknesses at 25 °C.

The uniaxial compressive stress–strain curves of the combined structure obtained at different temperatures or RPUF thickness ratios are shown in Figure 5. The stress–strain curve of the RPUF–SRF–FPS composite structure indicates that at lower strain levels, the composite is in the plastic deformation phase, achieving significant strain values with minimal increases in stress. As the internal structure of the composite undergoes compression and densification, the foam transforms into a dense reinforcement phase, resulting in a rapid increase in stress.

Furthermore, the mechanical properties of the RPUF–SRF–FPS composite structure exhibit a significant dependence on temperature and thickness. This is hypothesized to arise from the variation in material characteristics of each individual layer with changes in temperature and thickness. Consequently, the construction of a constitutive model that is capable of accurately predicting the stress–strain response of the composite structure necessitates conducting further investigations into the relationship between the elastic moduli of RPUF, SRF, and FPS with respect to temperature and thickness.

**Figure 6:**
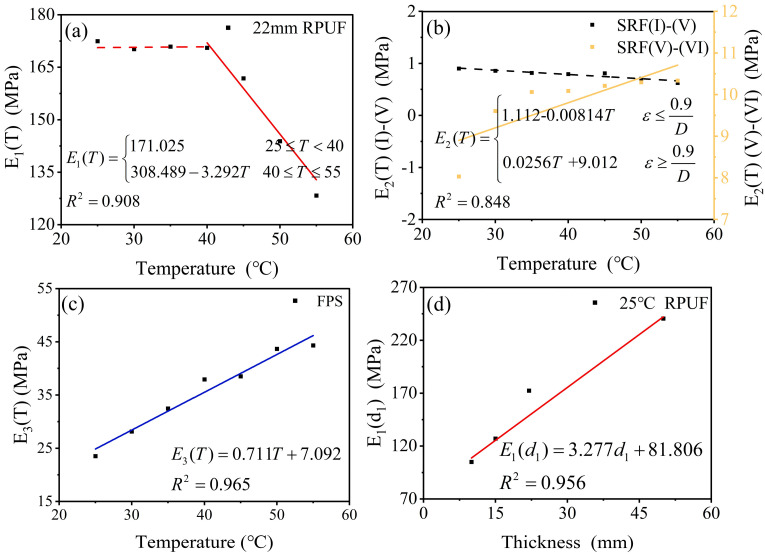
The modulus of elasticity of each monolayer is affected by temperature and thickness. (**a**) The 22 mm RPUF *E*_1_(*T*) curve with temperature. (**b**) SRF *E*_2_(*T*) curve with temperature. (**c**) FPS *E*_3_(*T*) curve with temperature. (**d**) RPUF *E*_1_(*d*_1_) curve with thickness.

The environmental temperature was altered using a temperature-controlled chamber, and quasi-static uniaxial compression tests were conducted to obtain the stress–strain data for RPUF, SRF, and FPS, as seen in Figures S1–S3 in the Supplementary Materials. For a monolayer material structure, σ=εE. Therefore, by further processing the experimental data obtained, the elastic moduli *E*_1_, *E*_2_, and *E*_3_ for RPUF, SRF, and FPS can be determined. The relationship between the temperature and the model parameters *E*_1_, *E*_2_, and *E*_3_ was established by fitting the stress–strain curves of each structural layer at varying temperatures, as illustrated in Figure 6.

As illustrated, the model parameters exhibit a pronounced dependence on the environmental temperature. The elastic modulus *E*_1_(*T*) of the RPUF remains relatively stable between 25 °C and 40 °C, but it shows a decline as the temperature increases from 40 °C to 55 °C (Figure 6a). This phenomenon can be attributed to the fact that 40 °C represents the glass transition temperature of RPUF, beyond which the material exhibits a notable softening effect. Conversely, it can be observed that the elastic modulus *E*_2_(*T*) of the SRF decreased slowly during the first stage and then increased rapidly when the strain exceeded a certain critical value depending on the temperature (Figure 6b). These phenomena are mainly attributed to the nonlinear deformation modes of the porous microstructure. The elastic modulus *E*_3_(*T*) of the FPS tends to increase with increasing temperature (Figure 6c). The FPS has two parts, including the reduced graphene oxide (rGO) coated polydimethylsiloxane mixture (PDMS) film with surface microstructures and a flexible interdigital electrode. In addition, PDMS behaves as a hyperelastic material, so the FPS exhibits material properties similar to those of the SRF.

Based on the experimental observations, the following equation can be derived by employing linear fitting of the model parameters:

(7) $$E_{1}(T)=\left\{\begin{array}{ll} 171.03 & \;25\leq T<40\\ 308.499\;-3.29\;T & 40\leq T\leq 55 \end{array}\right.$$

(8) $$E_{2}(T)=\left\{\begin{array}{ll} 1.11-0.0081T & \varepsilon \leq \frac{0.9}{D}\\ 0.26T+9.01 & \varepsilon \geq \frac{0.9}{D} \end{array}\right.$$

(9) $$\begin{array}{cc} E_{3}\left(T\right)=0.71T+7.09 & 25\leq T\leq 55 \end{array}$$

The unit of measurement of the numerical coefficients in these equations is MPa/°C.

To investigate the impact of RPUF thickness on composite structures, four distinct conditions were designed, with RPUF thicknesses of 10 mm, 15 mm, 22 mm, and 50 mm, and the stress–strain data are shown in Figure S4 of the Supplementary Materials. As illustrated in Figure 6d, the deformation behavior of RPUF’s elastic modulus at ambient temperature reveals a decreasing trend in elastic modulus with increasing RPUF thickness. This observation allows for fitting and adjustment of the constitutive model parameters, resulting in the following equation:

(10) $$E_{1}\left(d_{1},T\right)=\left\{\begin{array}{ll} 3.71d_{1}+78.84 & 25\leq T<40\\ 146.57+3.69\;d_{1}-1.78\;T & 40\leq T\leq 55 \end{array}\right.$$

The coefficients 3.71 and 3.69 have units of MPa/mm, while the coefficient 1.78 has units of MPa/°C.

Equation (7) is derived from experimental data for a 22 mm thickness RPUF layer, while Equation (10) provides a general form for varying thicknesses. The slight differences in coefficients are attributed to experimental uncertainties and the fitting process. To reflect practical accuracy, coefficients are rounded to two decimal places. By substituting Equations (8)–(10) into Equation (6), it is possible to further refine and enhance the constitutive model.

### 3.3. Model Verification

**Figure 7:**
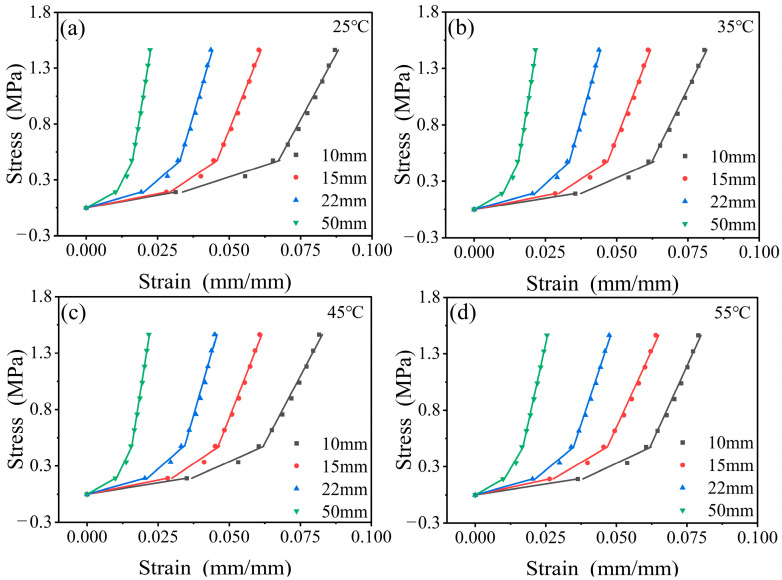
Constitutive model fitting of combinatorial structures with different RPUF thicknesses at different temperatures: (**a**) 25 °C, (**b**) 35 °C, (**c**) 45 °C, (**d**) 55 °C.

To verify the effectiveness of the established constitutive models of viscoelastic–hyperelastic composite structures in describing the compressive behavior, the stress–strain curves are calculated at a service temperature range of 25–55 °C. The predicted results are shown in Figure 7.

**Figure 8:**
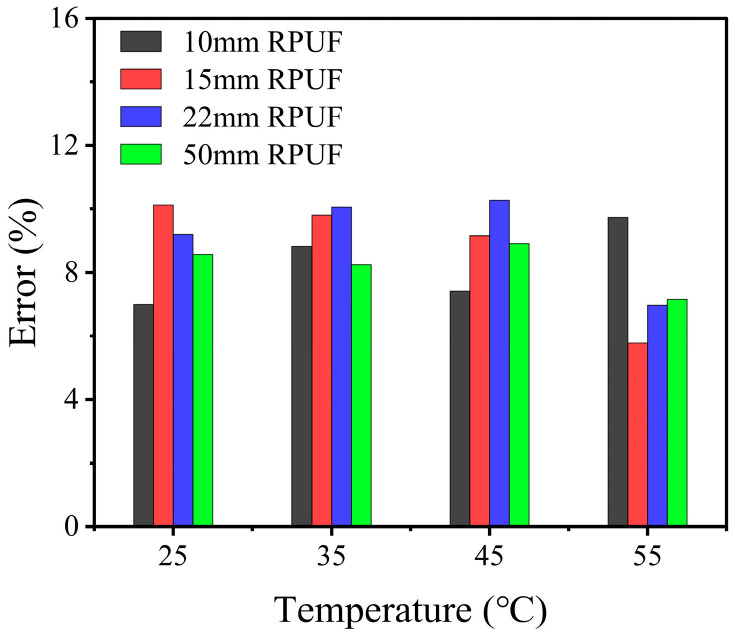
Constitutive model fitting of combinatorial structures with different RPUF thicknesses at different temperatures.

The predictive results exhibit a strong correlation with the experimental data, indicating that the developed constitutive model effectively forecasts the mechanical behavior of viscoelastic–hyperelastic composite structures. The discrepancies between the fitted stress–strain curves of the composite structures under varying temperature conditions and the experimental data are illustrated in Figure 8, with an average error of 8.6%. At this point, the constitutive model has been fully established. However, it is essential to emphasize that the derived model and its associated parameters are specifically formulated for uniaxial compression conditions and applicable solely under these circumstances. The applicability of this model to other stress states requires further investigation to ascertain its feasibility and robustness in broader contexts.

## 4. Application of Stress Monitoring Systems

### 4.1. Analysis of Pressure Monitoring Results

Based on the compositional model established in previous research, the relationship between the force *F* applied to the viscoelastic–hyperelastic composite structure and the force *F*_1_ exerted on the pressure monitoring system is represented as follows:

(11) $$\left\{\begin{array}{cc} F=\frac{A}{A_{1}}F_{1} & 0\leq F_{1}\leq 0.4\\ F=\left[1+\frac{A_{2}(\alpha +\beta +\gamma )}{A_{1}(\alpha +\beta )}\right]F_{1} & F_{1}\leq 4 \end{array}\right.$$

The pressure monitoring system indicates an inverse correlation between the applied force *F*_1_ and the elastic moduli *E*_1_, *E*_2_, and *E*_3_ of RPUF, SRF, and FPS, respectively.

**Figure 9:**
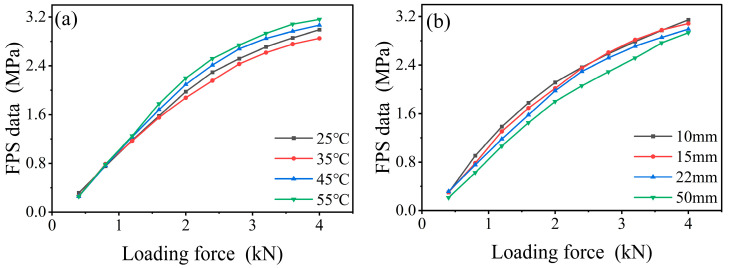
Monitoring results of ammunition surface pressure under the combined structure. (**a**) The 22 mm RPUF combined structure’s pressure sensor indication under different temperature conditions. (**b**) Indication of the pressure sensor under the combined structure of 25 °C with different RPUF thicknesses.

To investigate the effects of temperature and dimensions on the pressure monitoring system within the RPUF–SRF–FPS composite structure, experiments were conducted, and the results are illustrated in Figure 9. The experimental results in Figure 9a indicate that the mechanical response of the pressure monitoring system slightly decreases with increasing temperature in the range of 25 °C to 35 °C, while in the range of 45 °C to 55 °C, the response increases with rising temperature. Figure 9b demonstrates that as the thickness proportion of RPUF in the composite structure increases, the mechanical response of the pressure monitoring system consistently decreases.

**Figure 10:**
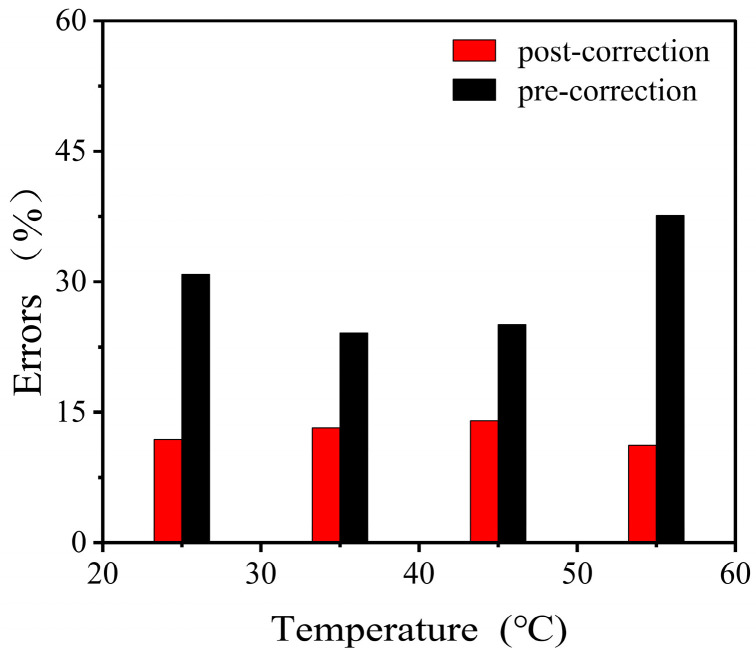
Measurement errors in pressure monitoring systems before and after correction of the constitutive model.

The experimental findings are in strong agreement with the validated constitutive model of the RPUF–SRF–FPS composite structure. This correlation indicates that the constitutive model possesses the ability to accurately predict the mechanical behavior of the RPUF–SRF–FPS composite structure under various loading conditions. Additionally, the error margin associated with the pressure monitoring system, which was meticulously calibrated in accordance with the parameters set forth by the constitutive model, exhibited a notable decrease from an initial value of 28% to a refined value of 12% (Figure 10). This significant reduction in error not only enhances the reliability of the pressure monitoring system but also provides critical insights that can inform the design and implementation of advanced multi-channel pressure monitoring systems within composite structures, thereby contributing to improved performance and safety in engineering applications.

### 4.2. Multi-Channel Pressure Monitoring System Applications

The findings from previous research indicate that the accuracy of the pressure monitoring system significantly improved following refinement of the constitutive model. Consequently, a multi-channel pressure monitoring system has been developed to broaden the monitoring scope and achieve comprehensive, multi-dimensional coverage, which has been subjected to practical application testing.

**Figure 11:**
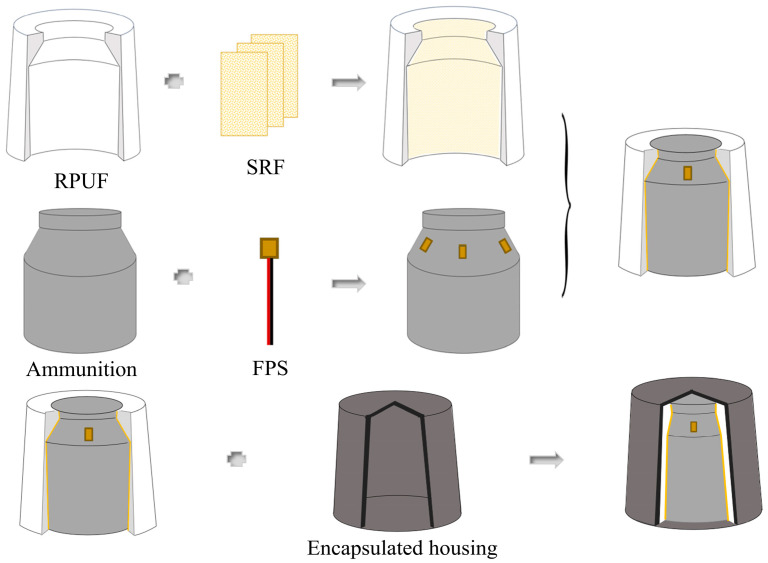
Schematic diagram of the practical application structure of the pressure monitoring system.

The FPS was uniformly affixed to the “shoulder” region of the ammunition, which endures the majority of the external loads. Subsequently, the RPUF–SRF protective structure was enveloped around the exterior of the ammunition where the FPS was adhered. Finally, the encapsulating shell was assembled, with the overall structural distribution illustrated in Figure 11. The monitoring system can accommodate up to 18 FPSs simultaneously, corresponding to the 18 channels within the acquisition module. The experiment utilized a composite structure of RPUF–SRF–FPS with a RPUF thickness of 30 mm, conducted at a temperature of 25 °C and an external loading force ranging from 0 to 10 kN.

**Figure 12:**
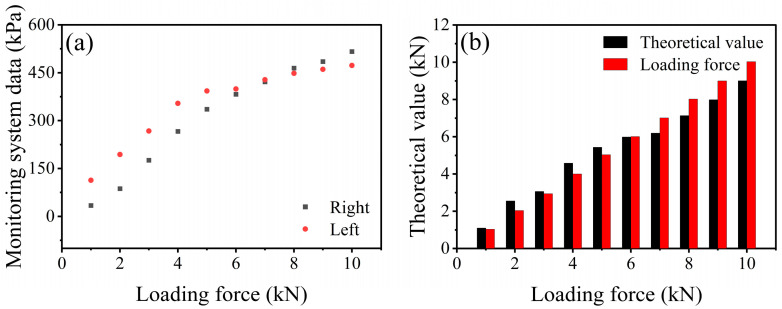
Actual loading force and theoretical calculation data. (**a**) Multi-channel pressure monitoring system data under loading force 0–10 kN. (**b**) Error between the theoretical correction data of the constitutive model and the actual loading force.

The experimental results are illustrated in Figure 12. From Figure 12a, it is evident that during the loading process of the ballast force, the initial stress monitoring data for the left half of the composite structure are significantly higher than those of the right half. However, as the force increases, the stress monitoring data for the right half gradually surpass those of the left half. This phenomenon can be attributed to the fact that the loading force is applied through the screws located above the external casing. By tightening the screws, the internal composite structure is elevated, creating a compressive interaction with the external casing, resulting in stress. The sequential rotation of the screws from left to right leads to uneven stress distribution within the composite structure. The multi-channel pressure monitoring system, after correction with the constitutive model, shows that its monitoring data correlate with the external loading force, as depicted in Figure 12b, with a monitoring error of 13%.

The practical application results of the multi-channel pressure monitoring system indicate that the pressure data recorded during the ammunition storage process are largely consistent with the actual pressure conditions. Verification through field experiments confirms that the pressure monitoring system currently meets the requirements for long-term monitoring of surface pressure on ammunition.

## 5. Conclusions

A constitutive model tailored to various polymer foam composite structures is adept at precisely characterizing mechanical behavior under quasi-static compression. Furthermore, the temperature dependencies of the material parameters for each distinct layer are established through experimental methodologies. It is suggested that the mechanical response of composite structures emerges from the collective mechanical behaviors of the individual layers, with the hyperelastic material SRF dominating the overall strain response under a uniform stress field. As the stress continues to increase, the influence of the viscoelastic material RPUF on the mechanical performance of composite structures becomes increasingly apparent, even when RPUF constitutes a larger proportion of the thickness within the composite structure. Additionally, a multi-channel pressure sensing system with a monitoring range of 8 MPa is employed, designed for pressure assessment in composite structures. From the insights gained through constitutive model research, the pressure monitoring system is optimized to guarantee measurement accuracy within a service temperature range of 25–55 °C. The sensing system is capable of delivering real-time visualizations of stress conditions at various locations within the composite structure. Its reliable performance renders it suitable for intricate environments, showcasing considerable application potential in the domain of structural health monitoring.

## Figures and Tables

**Table 1:** Dimensions of compression test specimens.

Style Number	Thickness (mm)	Diameter (mm)
1	10	60
2	15	60
3	22	60
4	50	60

## Data Availability

The original contributions presented in this study are included in the article. Further inquiries can be directed to the corresponding author.

## Supplementary Materials

The following supporting information can be downloaded at: https://www.mdpi.com/article/10.3390/polym17050647/s1, Figure S1: Stress-strain curves of 22 mm RPUF at different temperatures. (a) 25–40 °C. (b) 40–55 °C; Figure S2: Stress-strain curves of SRF at different temperatures; Figure S3: Stress-strain curves of FPS at different temperatures; Figure S4: Stress-strain curves of RPUF with different thicknesses at 25 °C.

## DURC Statement

We have made the following statements in our paper titled “Investigation of constitutive models for pressure monitoring of viscoelastic-hyperelastic composite structures”.

- Explanation of Potential Risks: Our paper investigates the mechanical properties of viscoelastic–hyperelastic material composite structures. This study is confined to providing theoretical and experimental support for the development of multilayer polymer material composite structures and does not pose any threat to public health or national security.
- Evaluation of Benefits to the General Public: Our research is limited to the academic field, which is beneficial to the development of material science. There is no risk to the general public.
- Compliance With Laws: As an ethical responsibility, we strictly adhere to relevant national and international laws about dual-use research. And, we have considered and adhered to these regulations in our paper.
