# Supplementary material for: Investigation of Constitutive Models for Pressure Monitoring of Viscoelastic–Hyperelastic Composite Structures
Source: Polymers (Basel). 2025 Feb 28;17(5):647. doi: 10.3390/polym17050647 (PMC11902559; doi:10.3390/polym17050647)
Supplement: Supplementary file 1 [file polymers-17-00647-s001.zip › polymers-3459167-supplementary.pdf]

polymers

Article

Investigation of Constitutive Models for Pressure Monitoring of Viscoelastic–Hyperelastic Composite Structures

Lijia Ai [1,2], Peng Li [2], Hongwei Yuan [1], Chunrong Tian [1], Xiaolian Qiang [1] and Tao Fu [1,*]

1 Institute of Chemical Materials, China Academy of Engineering Physics, Mianyang 621900, China; 20181985@s.hlju.edu.cn (L.A.); yuanhw@caep.cn (H.Y.); tianchr_icm@caep.cn (C.T.); qiang.xiaolian@caep.cn (X.Q.)
2 College of Physical Science and Technology, Heilongjiang University, Harbin 150080, China; lipenghit@hlju.edu.cn
* Correspondence: futao@caep.cn

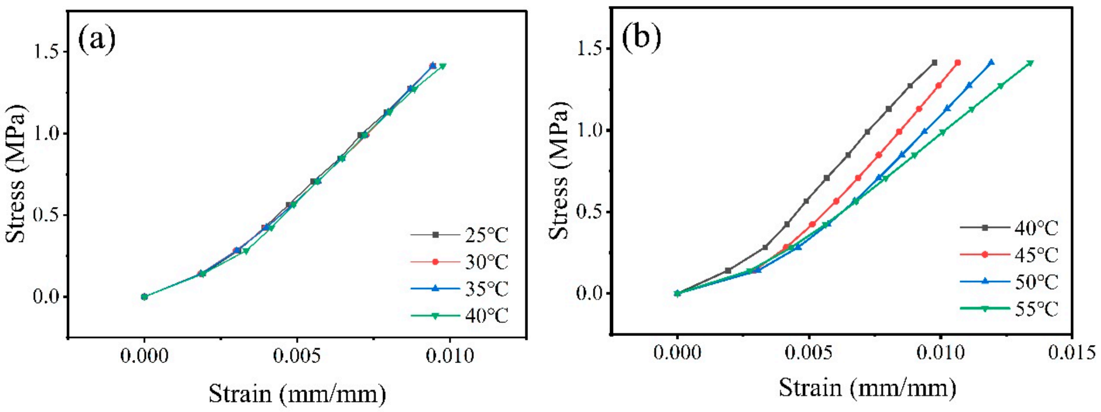

Figure S1. Stress-strain curves of 22mm RPUF at different temperatures. (a).25-40°C. (b).40-55°C.

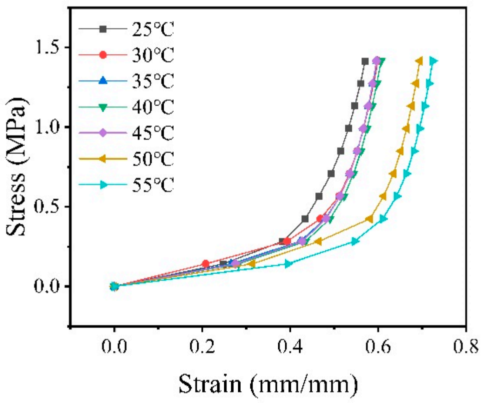

Figure S2. Stress-strain curves of SRF at different temperatures.

 https://doi.org/10.3390/polym17050647

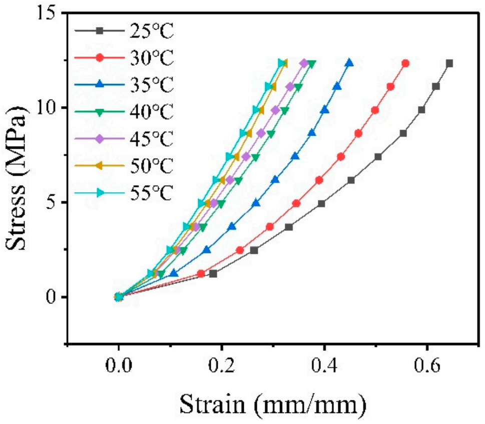

Figure S3. Stress-strain curves of FPS at different temperatures.

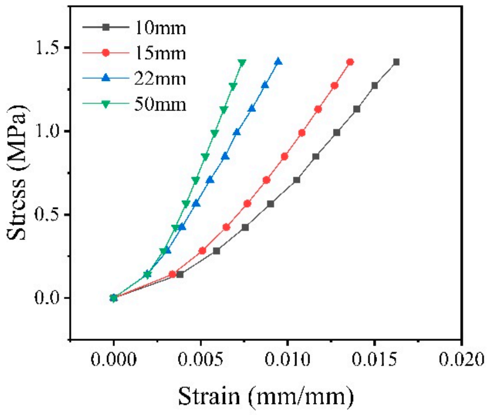

Figure S4. Stress-strain curves of RPUF with different thicknesses at 25°C.
